# Advancing Needle-Free Jet Injectors for Global Vaccine Delivery

**DOI:** 10.3390/pharmaceutics18040417

**Published:** 2026-03-28

**Authors:** Peter Ikechukwu, Remigius Agu

**Affiliations:** 1 Department of Pharmacology and Toxicology, Faculty of Pharmaceutical Sciences, University of Nigeria, Nsukka 410001, Enugu State, Nigeria; 2 Biopharmaceutics and Drug Delivery Lab, Faculty of Health, College of Pharmacy, Dalhousie University, P.O. Box 15000, Halifax, NS B3H 4R2, Canada

**Keywords:** needle-free jet injectors, vaccine delivery, regulatory harmonization, immunogenicity, pandemic preparedness, self-administration, public–private partnerships

## Abstract

**Background**: Global immunization programs continue to rely on needle-based injections despite persistent concerns regarding sharps disposal, accidental injuries, and the technical skill required for accurate intradermal administration. Needle-free jet injectors (NFJIs) are an alternative delivery method in which narrow, high-velocity liquid jets penetrate the skin without a needle. Contemporary designs, ranging from single-use disposable-syringe injectors to digitally controlled electromechanical devices, address historical safety issues and meet current WHO and FDA device expectations. **Methods**: Evidence from engineering analyses, preclinical modeling, and clinical trials was reviewed to characterize how jet velocity, nozzle structure, and formulation rheology influence skin penetration and drug dispersion. Published vaccine studies were examined for antibody responses, seroconversion, and reactogenicity compared with needle–syringe injection. Field vaccination campaign data from national campaigns and operational reports were evaluated to describe implementation steps, acceptability, and implementation constraints. **Results**: Published studies evaluating vaccines, including inactivated influenza, hepatitis B, typhoid, rabies, and measles, report antibody titers and seroconversion rates after NFJI administration that are comparable to those achieved with conventional intramuscular or intradermal needle injection. Needle-free delivery was associated with operational advantages in several immunization programs, including reduced sharps waste and improved vaccination rate during high-volume immunization campaigns. Local and systemic reactogenicity follows expected patterns, with slightly higher injection-site responses in some NFJI studies. Imaging and mechanical data confirm that jet performance depends on nozzle geometry and controlled pressure pulses. At the same time, formulation stability remains a critical determinant of successful jet-based vaccine administration, particularly for protein antigens, adjuvanted formulations, and emerging mRNA vaccines that may experience transient shear stress during high-velocity injection. Evidence from vaccination campaigns further indicates that needle-free jet injectors reduce sharps waste, simplify vaccine handling and administration procedures, and support rapid vaccine delivery in large-scale immunization programs. **Conclusions**: Needle-free jet injectors are a practical alternative to traditional needle-based injections for some vaccines. Their main benefits include enabling intradermal dose-sparing strategies, reducing reliance on sharps disposal methods, and enabling the efficient vaccination of large groups without compromising immunogenicity. Future research should define the physicochemical stability limits of biologic formulations subjected to jet injection and evaluate digitally controlled injectors capable of precise pressure modulation and adjustable delivery parameters. In addition, needle-free jet injection eliminates needle penetration and sharps handling, which may reduce needle-associated anxiety and improve vaccine acceptability among individuals with needle aversion.

## 1. Introduction

### 1.1. Evolution of Needle-Free Jet Injection

Propelling a narrow stream of liquid into the skin without a needle is a concept that predates modern vaccination programs. Early mechanical jet injectors appeared during global smallpox eradication campaigns, when vaccination programs required high-volume delivery while seeking to reduce accidental needlestick injuries among health workers [1]. These devices relied on spring-loaded or gas-driven mechanisms that generated brief high-velocity bursts capable of depositing vaccine into dermal or subcutaneous tissues [2,3]. Although effective for specific campaigns, these early injectors lacked the precision, safety safeguards, and compatibility controls expected today, limitations highlighted in later evaluations of multi-use nozzle injectors [4].

**Figure 1:**
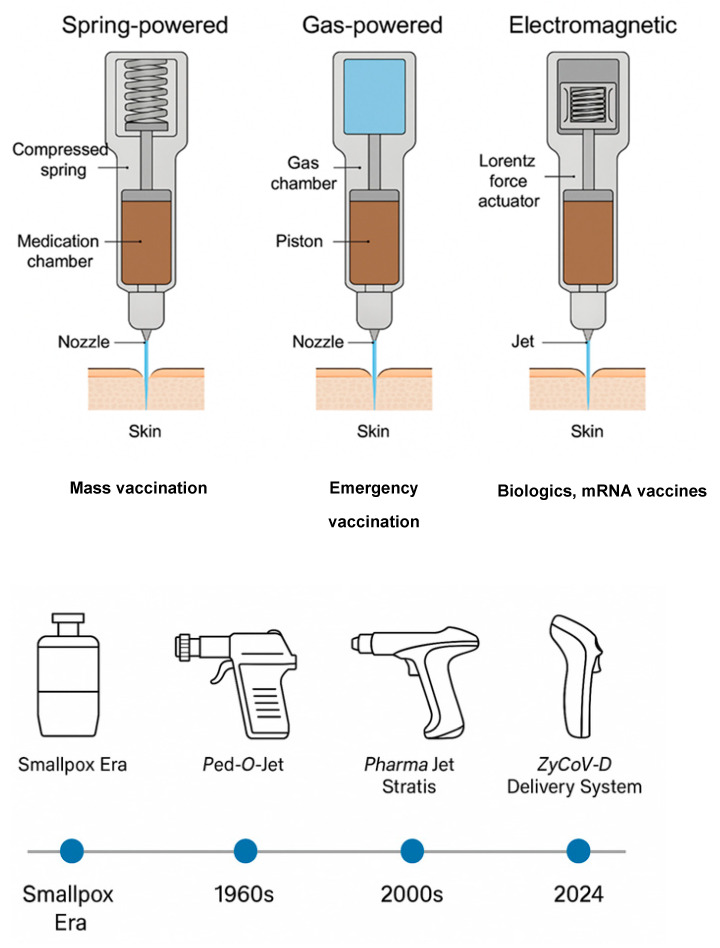
Evolution of Needle-Free Jet Injectors Across Four Technological Eras.

As childhood immunization expanded during the 1980s and 1990s, attention shifted toward disposable needle–syringe injectors. These devices were inexpensive, easy to train on, and compatible with most formulations. However, dependence on metal needles raised ongoing safety issues. Reports documenting unsafe injections and improper disposal described occupational injuries across vaccination programs [5,6], while community perceptions of sharps risk persisted despite improvements in sterility practices [7]. These concerns sustained interest in delivery technologies capable of reducing sharps use while maintaining reliability. The technological progression of needle-free jet injectors, from early mechanical systems to modern disposable-syringe and digitally controlled injectors, is summarized in Figure 1.

Subsequent engineering improvements revived interest in jet injection technology. Disposable-syringe jet injectors (DSJIs) incorporated single-use fluid pathways, controlled pressure profiles, and more predictable penetration depths [8]. Clinical studies of intradermal administration demonstrated that high-velocity fluid jets could achieve reliable dermal dispersion while eliminating cross-contamination risks associated with earlier multi-use nozzles [9,10]. These advances coincided with heightened expectations for safety-engineered vaccine-delivery devices and expanded technical guidance for injection devices used in immunization programs [11,12,13].

Several additional factors have contributed to renewed attention to needle-free jet injection technologies. Global demand for vaccines continues to increase, while biologic formulations have become more complex, and many health systems face shortages of trained vaccinators. In many regions, vaccination programs must immunize large populations despite limited availability of trained personnel and limited time for device-specific training. Reliable sharps disposal infrastructure is also inconsistent in some settings, as documented in WHO program assessments [14,15]. At the same time, anxiety associated with needle-based injection remains common among children and adults [16,17,18].

Interest in jet injections is further supported by the immunological advantages of intradermal delivery. Several vaccines and biologics generate strong immune responses when administered into the dermis, but manual intradermal injection requires technical skill and consistent technique. Jet injectors generate a narrow, controlled plume that spreads across the dermal plane and supports intradermal dose-sparing strategies evaluated for influenza, polio, rabies, dengue, and other pathogens [8,13,19]. Dose-sparing approaches are particularly important for supply-limited vaccines, as noted in WHO reports on temperature sensitivity and controlled-temperature-chain strategies [20,21].

Recent studies published between 2020 and 2025 further reflect renewed research activity focused on improving vaccine delivery efficiency, formulation compatibility, and scalability for large immunization programs [22,23,24,25]. Countries that have expanded vaccination coverage in recent decades now face increasingly complex operational demands, including multi-dose schedules, catch-up campaigns, and emergency vaccination efforts. Jet injectors reduce sharps waste and simplify the administration sequence by removing steps associated with needle attachment and disposal. Evidence from national vaccination campaigns indicates strong community acceptance when devices are introduced with transparent communication addressing concerns about pain and needle anxiety [22,23]. These operational advantages make needle-free jet injectors a practical complement to conventional needle–syringe injection. Progressive evolution from early mechanical jet injectors (1940s–1960s), to gas-powered devices, to DSJIs with single-use sterile pathways, and to modern electromechanical systems with digitally controlled actuators are summarized in Figure 1. Representative mechanisms, expected delivery depths, and operational characteristics, emphasizing progress in sterility assurance, precision control, and compatibility with shear-sensitive biologics, are compared.

### 1.2. Scope and Structure of This Review

This review examines needle-free jet injectors (NFJIs) as vaccine delivery devices in which injector engineering, formulation characteristics, and vaccination program operations determine clinical and operational performance. Instead of viewing jet injection solely as a mechanical alternative to needles, the discussion places NFJIs within the broader pharmaceutics context and examines how fluid dynamics, nozzle geometry, and actuation profiles influence biologics stability and immunogenic outcomes. The first sections describe the physical principles governing jet injection, including jet formation, skin penetration mechanics, and energy sources responsible for pressure generation and control. These concepts highlight how NFJIs achieve reliable intradermal and intramuscular delivery and how injector design influences dispersion patterns, penetration depth, and safety.

Subsequent sections examine formulation and biologics considerations, including rheology, viscosity, and the response of proteins, viral vectors, and nucleic-acid vaccines to high-velocity shear and acceleration. Clinical experience with licensed vaccines delivered by NFJIs is then summarized, with attention to immunogenicity, dose-sparing strategies, reactogenicity, and patient acceptance compared with conventional needle–syringe injections. Experiences from national vaccination programs and emergency immunization campaigns are also considered to highlight operational efficiency, training requirements, and community perception. The review concludes with a discussion of regulatory, economic, and environmental considerations and identifies research priorities likely to influence the next generation of jet injection technologies and their role in global vaccination efforts.

## 2. Physical Principles of Needle-Free Jet Injection

### 2.1. Jet Formation and Fluid Dynamics

Jet injection relies on the rapid conversion of stored mechanical or pneumatic energy into a high-velocity liquid stream capable of penetrating the skin barrier. When pressure is released through a narrow orifice, the formulation accelerates to velocities typically exceeding 100 m/s, forming a coherent jet that behaves differently from liquid flow under conventional injection conditions. The characteristics of this jet, such as velocity profile, coherence, and breakup behavior, are governed by nozzle geometry, pressure rise time, and the physical properties of the formulation, including density and viscosity [9,24]. The major NFJI designs, their energy sources, delivery depths, and practical limitations are summarized in Table 1.

Experimental and modeling studies describe jet formation as a transient process in which the initial pressure pulse establishes a leading jet front, followed by a trailing column that sustains penetration and dispersion. High-speed imaging and penetration studies indicate that the dimensions of this plume are not arbitrary but fall within measurable ranges that vary with injector design and target tissue. In experimental skin models, intradermal jet injection typically produces penetration depths of approximately 2–5 mm, whereas devices configured for deeper intramuscular delivery may reach 5–12 mm, depending on pressure, nozzle diameter, and pulse duration. Lateral spread within the dermis often extends several millimeters from the point of entry, helping explain the characteristic fan-shaped or planar dispersion pattern observed after successful jet administration. This distribution arises because, once the jet breaches the stratum corneum and epidermis, fluid preferentially propagates along dermal tissue planes and interfaces of lower mechanical resistance and does not necessarily continue as a narrow linear tract [7,8,9,13,15,16]. Short, sharp pressure ramps tend to produce highly focused jets with minimal lateral spread at the skin surface. In contrast, slower or irregular pressure profiles can lead to jet instability, plume widening, or premature breakup [8,10]. These effects influence not only penetration depth but also the uniformity of intradermal or subcutaneous distribution.

Fluid dynamic analyses further show that nozzle diameter plays a critical role in balancing penetration and safety. Smaller orifices generate higher jet velocities at a given pressure but increase shear stress within the fluid. In comparison, larger orifices reduce shear but may require higher total energy to achieve sufficient penetration [7,13]. Computational and high-speed imaging studies have demonstrated that optimal jet performance depends on matching orifice size and pressure-time profiles to the target tissue layer and formulation characteristics, and not by maximizing velocity alone [22].

These findings establish jet formation as a controllable but sensitive process. Small variations in actuation or nozzle design can produce measurable differences in penetration efficiency, dispersion geometry, and tissue response. Understanding these fluid dynamic principles is therefore essential for interpreting both preclinical performance data and clinical outcomes associated with different NFJIs.

### 2.2. Skin Penetration Mechanics

**Figure 2:**
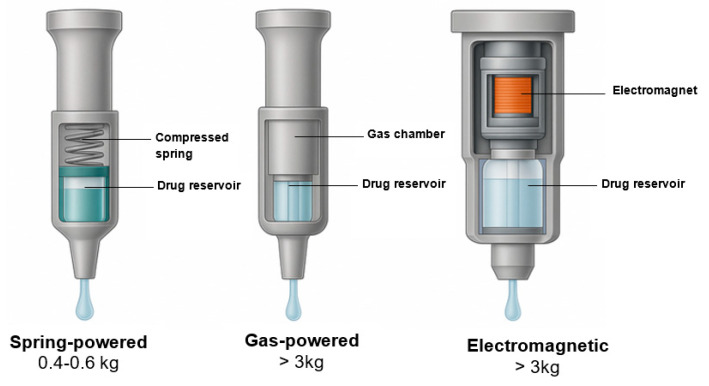
Comparative Design Features of Spring-Powered, Gas-Powered, and Electromagnetic NFJIs. Adapted from [2,27], published by Elsevier, 2016 and Elsevier, 2014. Cross-sectional comparison of spring, gas, and electromagnetic configurations highlighting actuator design, energy release kinetics, nozzle geometry, and dispersion patterns. Electromagnetic systems enable real-time determination of jet velocity and pressure-rise profiles. Spring systems favor simplicity; gas systems support adjustable energy with trade-offs in logistics; electromagnetic systems enable sub-millimeter precision and digital add-ons, which are advantageous for field vaccination campaign choices for dose-sparing and biologics compatibility.

The ability of a liquid jet to cross the skin barrier depends on the relationship between jet velocity, orifice diameter, and the duration of the pressure pulse. Reliable penetration of the epidermis requires the jet to exceed a threshold momentum sufficient to overcome the mechanical resistance of the stratum corneum and underlying tissue layers. Experimental studies using ex vivo skin models and in vivo imaging demonstrate that when this threshold is not reached, jets disperse superficially or rebound from the skin surface, resulting in incomplete or inconsistent delivery [7,10]. Once the jet breaches the epidermis, penetration behavior is governed by both fluid properties and tissue structure. Unlike solid needles, which form a linear tract, liquid jets create a narrow entry point that rapidly expands laterally within the dermis, producing a fan-shaped or planar dispersion pattern. High-resolution imaging of intradermal administration shows that this lateral spread follows natural tissue planes and connective structures, promoting relatively uniform antigen distribution when pressure profiles are appropriately controlled [8]. Jet pressure and pulse duration jointly determine the depth reached before dispersion occurs. Excessive force may drive the jet beyond the intended layer, increasing the likelihood of subcutaneous deposition or tissue trauma, whereas insufficient force may lead to shallow placement or leakage. Modeling studies linking pressure profiles to tissue deformation indicate that modest changes in pulse duration can shift deposition depth by several millimeters [13,25]. Structural and actuation differences between spring-powered, gas-powered, and electromechanical NFJIs, and their implications for velocity control and dispersion behavior, are summarized in Figure 2.

These mechanical factors also influence tissue response among different age groups. Although modern NFJIs are designed to minimize trauma, excessive penetration force or poorly calibrated pressure settings may contribute to localized erythema, bruising, petechiae, or transient capillary disruption, particularly when injections extend beyond the intended dermal plane. Such effects may be more pronounced in populations with distinct skin properties, including young children, whose skin thickness and dermal composition differ from those of adults, and older adults, in whom reduced elasticity and increased capillary fragility may affect tolerability. These age-related differences underscore the importance of selecting appropriate jet pressure, nozzle geometry, and pulse duration for the target population [4,8,15,21,33,34]. Clinical and operational observations further support the importance of these mechanical principles. Consistent penetration depth is associated with predictable reactogenicity profiles and reduced variability in immune responses, whereas erratic penetration may contribute to pain, leakage, or incomplete dosing. Device evaluation studies indicate that consistent actuation, stable nozzle performance, and single-use fluid pathways are critical for reproducible penetration depth and reliable dose delivery in different patient populations and vaccine formulations [11,22,26].

### 2.3. Energy Sources and How Actuation Affects Delivery

Needle-free jet injectors employ different energy sources to generate the pressure required for jet formation, and these choices directly influence actuation profiles, reproducibility, and device performance. Early systems relied primarily on mechanical springs or compressed gas, resulting in rapid pressure release but limited control over pulse shape and duration. While these approaches were sufficient for high-volume vaccination programs, variability in pressure output and wear-related drift constrained precision and long-term reliability [2,3].

In modern disposable syringe jet injectors, earlier concepts have been refined by using calibrated springs, regulated gas cartridges, or hybrid propulsion systems, which result in more predictable pressure–time profiles. Controlled actuation ensures that each injection produces a consistent pressure pulse, reducing variability in jet velocity and penetration depth across repeated uses. Studies evaluating these injectors demonstrate that standardized injector actuation improves dose-delivery accuracy and contributes to more consistent intradermal dispersion, particularly during vaccination of large numbers of recipients [8,11]. Electromechanical systems extend this technological progression by using motors and digital control systems to dynamically regulate jet pressure during injection. These devices can adjust acceleration ramps, peak pressure, and pulse duration in real time, allowing finer tuning of jet characteristics for different target tissues or formulation properties. Engineering analyses indicate that such control can reduce peak shear stress while maintaining sufficient penetration, a feature of growing importance for shear-sensitive biologics and nucleic-acid-based vaccines [12,24].

Among propulsion injector types, effective jet injection depends not only on achieving adequate peak pressure but also on the temporal profile of energy release. Abrupt, poorly controlled pulses increase the risk of jet instability and tissue trauma, whereas smoothly regulated actuation supports coherent jet formation and predictable dispersion. As NFJI technology continues to evolve, the selection and optimization of energy sources remain central to balancing mechanical performance, biologic compatibility, and operational robustness.

### 2.4. Safety, Nozzle Design, and Cross-Contamination Prevention

Safety considerations have been central to the redesign of needle-free jet injectors following concerns raised during early multi-use-nozzle deployments. Historical systems demonstrated that inadequate barriers between injections could permit retrograde flow of biological material, creating a risk of cross-contamination when devices were reused between individuals [4]. These findings prompted a shift toward designs that physically isolate the fluid pathway for each injection and eliminate direct contact between the nozzle and the skin.

These risks are addressed by using contemporary single-use disposable components in NFJIs, including cartridges, syringes, and nozzle assemblies, which are discarded after each injection. Such designs prevent backflow and eliminate the need for between-patient sterilization, thereby supporting compliance with modern infection-control standards. Laboratory and field evaluations confirm that these systems effectively prevent cross-contamination when used in accordance with manufacturer specifications [9,10,11]. Nozzle geometry also plays a role in both safety and performance. Precisely engineered orifices maintain jet coherence while minimizing splash-back and surface contamination at the skin interface. Some designs incorporate recessed or shielded nozzles that reduce direct contact with the injection site, further limiting the potential for contamination while also stabilizing the jet during initial skin contact [28,29]. These features contribute to consistent penetration mechanics and reduce variability in delivery outcomes.

Regulatory agencies now explicitly require verification of single-use safeguards, biocompatibility of materials, and validation of pressure and flow performance across the device’s operating range. Standards and guidance from WHO, FDA, and ISO emphasize risk management, traceability, device verification, and post-market surveillance to ensure that safety features remain effective under real-world conditions [26,30,31,35,36]. These requirements have helped reposition NFJIs as delivery devices that meet contemporary expectations for mechanical performance and infection control.

## 3. Formulation and Biologic Considerations

### 3.1. Rheology, Viscosity, and Jet Stability

Formulation rheology is a critical determinant of jet stability and penetration behavior during needle-free injection. As a formulation accelerates through a narrow orifice under high pressure, its viscosity and flow characteristics influence whether a coherent jet forms or whether the stream fragments prematurely. Low-viscosity solutions generally form narrow, high-velocity jets that penetrate efficiently. In contrast, higher-viscosity formulations require greater driving force to achieve comparable penetration, altering the pressure–time profile needed for reliable delivery [30,32].

Experimental studies have shown that viscosity affects both jet coherence and dispersion geometry within the skin. At moderate viscosities, jets remain cohesive and spread laterally within the dermis as intended. At very low levels of viscosities, however, the jet may disperse too rapidly after entry, increasing variability in deposition patterns. Conversely, highly viscous formulations can resist acceleration, leading to reduced penetration depth or increased surface splash-back if pressure is not adequately matched to formulation properties [32,37].

Jet stability is also influenced by non-Newtonian behavior, which is increasingly relevant for biologic formulations containing polymers, adjuvants, or lipid-based carriers. Shear-thinning formulations may exhibit transient viscosity reduction during injection, facilitating penetration but complicating prediction of dispersion once shear forces dissipate. These effects highlight the importance of characterizing formulation rheology under conditions that approximate the high shear rates encountered during jet injection as opposed to relying solely on low-shear laboratory measurements [30,38]. These findings indicate that optimal NFJI performance depends on the compatibility between formulation rheology and the injector actuation parameters. Appropriate combinations of viscosity, nozzle geometry, and pressure profiles promote stable jet formation, predictable penetration, and consistent intradermal or subcutaneous dispersion, forming the basis for reliable delivery of biologic vaccines.

### 3.2. Biologics Integrity Under Jet Injection

The delivery of modern vaccines via needle-free jet injectors raises important questions about the structural integrity of biologics under rapid pressurization, high shear, and acceleration. Many contemporary vaccines rely on macromolecular structures (recombinant proteins, viral vectors, or nucleic-acid-based vaccines) that are inherently sensitive to physical stress. During jet injection, these materials experience transient shear gradients and pressure changes that differ substantially from those encountered during conventional needle-syringe delivery [20,39].

Protein-based vaccines have generally demonstrated acceptable stability under NFJI conditions when formulations are optimized, and pressure profiles are well controlled. Studies examining subunit and inactivated vaccines report preserved antigenicity and comparable immunogenicity following jet injection, suggesting that most protein antigens tolerate short-duration mechanical stress without significant denaturation or aggregation [8,12]. However, susceptibility varies with protein structure, buffer composition, and the presence of stabilizing excipients. Thus, emphasizing the need for formulation-specific evaluation.

Nucleic acid-based vaccines, including mRNA and DNA, are inherently difficult to deliver via NFJIs because of their complexity. These vaccines often rely on lipid-based carriers or other delivery vehicles that can be disrupted by shear or cavitation. Published data indicated that while some lipid nanoparticle formulations withstand jet injection without measurable loss of potency, others exhibit changes in particle size distribution or encapsulation efficiency under aggressive pressure profiles [9,21,24]. These findings underscore the importance of selecting injector actuation parameters that remain within the shear tolerance of the formulation, thereby preserving antigen structure and biological activity. Beyond shear exposure alone, the extremely rapid acceleration and deceleration during jet formation may create localized stress environments that differ from those encountered with conventional needle-based injections. High-pressure gradients and short-duration extensional flow can influence protein conformation, lipid nanoparticle integrity, and viral vector stability, particularly when formulations are not optimized for high-velocity delivery. Experimental evaluations using model proteins have shown that while most vaccines tolerate these stresses, especially when pressure profiles are properly controlled, excessive jet energy or poorly matched nozzle geometry can increase the likelihood of transient aggregation or structural perturbation. These observations reiterate the importance of combining formulation development with device engineering to preserve biologics stability under realistic injection conditions [17,18,19,40]. Within this broader context, viral-vectored vaccines are a distinct class of biologics whose structural robustness and mechanical sensitivity must be evaluated separately. Available evidence suggests that many vectors remain stable during NFJI delivery, but performance depends on vector type, formulation viscosity, and nozzle geometry [30,39]. These observations indicate that biologic formulations do not respond uniformly to jet injection; successful delivery depends on appropriate combinations of formulation composition, injector design, and actuation parameters that preserve vaccine stability and immunogenic potential.

### 3.3. Biologics Stressors—Shear, Aggregation, and Degradation

Formulations delivered by needle-free jet injectors are exposed to a distinct set of mechanical stressors that can influence biologic stability. As liquid accelerates through a narrow nozzle under high pressure, it encounters intense shear forces, rapid pressure transitions, and transient extensional flow. These conditions differ significantly from those experienced during conventional needle injection and can affect macromolecular structure, particularly for complex or multicomponent vaccine formulations [30,39].

Shear stress is a primary concern for proteins and particulate systems. High shear gradients can promote partial protein unfolding, increasing the risk of aggregation or loss of functional epitopes. While many licensed vaccines are resilient to brief shear exposure, studies have shown that certain adjuvanted or highly structured formulations are more vulnerable, particularly when pressure profiles generate turbulent flow or cavitation near the nozzle exit [32,38,41]. Aggregation not only reduces antigen availability but may also alter immunogenicity or increase reactogenicity. Rapid decompression following jet formation can induce localized cavitation or interfacial stress, especially in formulations containing surfactants or lipid-based carriers. These effects have been implicated in changes to particle morphology and stability for lipid nanoparticle formulations used in nucleic-acid vaccines [9,21,24]. Although such changes may be subtle, they can influence delivery efficiency and downstream immune responses.

Addressing these stressors requires testing strategies that reflect realistic injection conditions. Conventional stability assays performed under static or low-shear conditions may fail to capture the transient stresses encountered during NFJI delivery. Increasingly, investigators employ pressure-driven flow models, high-shear rheometry, and device-coupled stress testing to evaluate formulation robustness under conditions that approximate actual jet injection profiles [22] These approaches support more informed co-development of formulations and devices, ensuring that the integrity of biologic drugs is maintained throughout high-velocity delivery.

## 4. Clinical Performance and Immunogenicity

### 4.1. Licensed Vaccines Delivered via NFJI

Clinical experience with needle-free jet injectors spans several decades and includes a range of licensed vaccines delivered through intradermal, subcutaneous, and intramuscular routes. Early and contemporary studies have evaluated NFJIs for inactivated, live-attenuated, and subunit vaccines, providing a foundation for assessing immunogenicity, safety, and operational feasibility relative to needle–syringe injection [3,27,42]. Comparative immunogenicity, seroconversion, and reactogenicity outcomes for licensed vaccines delivered by NFJIs versus needle–syringe administration is summarized in Table 2.

Across multiple vaccine classes, antibody responses elicited by NFJI delivery are generally comparable to those achieved with conventional injections. Studies involving inactivated influenza vaccines demonstrated similar seroconversion rates and geometric mean titers following intradermal or intramuscular jet injection, supporting the use of NFJIs in both routine and campaign settings [8,13]. Comparable findings have been reported for hepatitis B, typhoid, rabies, and measles-containing vaccines, where immune outcomes meet established non-inferiority criteria [34,35,36,43,44,45].

Safety profiles observed in these trials are in agreement with expectations for the respective vaccines. Local reactogenicity, including erythema, induration, and tenderness at the injection site, is frequently reported and, in some cases, occurs at slightly higher rates following intradermal jet injection. These reactions are typically mild and self-limited, resolving without intervention. Systemic adverse events mirror those seen with needle–syringe delivery, indicating that jet injection does not involve new systemic safety concerns [12,22,46]. Evidence from vaccination programs further supports these clinical findings. Large-scale mass immunizations using disposable-syringe jet injectors demonstrate reliable dose delivery and consistent performance across large populations. These data indicate that, across a broad range of licensed vaccines, NFJIs are associated with immunogenicity and safety outcomes comparable to traditional injection methods and offer advantages in vaccine administration, including device preparation, dose loading, injection delivery, and disposal of consumables.

**Table 2:** Comparative Immunogenicity and Reactogenicity of Vaccines Delivered by NFJI vs. Needle–Syringe.

Vaccine/Platform	Study/Setting	Age Group	*N*	Immunologic Endpoint	Comparator	GMT ratio (NFJI:NS)	Seroconversion % (NFJI vs. NS)	Local AE % (NFJI vs. NS)	Systemic AE % (NFJI vs. NS)	NI/Outcome	Device (route)	Citation(s)
Inactivated influenza (TIV/QIV)	Randomized IM trial; high-income	Adults	600–1000	HI GMT; seroconversion day 21–28	Standard IM NS	0.9–1.2	60–80 vs. 60–82	35–55 vs. 30–50	20–35 vs. 18–32	NI met; increased local AEs	Spring/gas DSJI (IM)	[26,27,41,42]
Vi capsular polysaccharide typhoid	Comparative clinical study	Adults	100–300	Serum IgG; GMT; seroconversions	Standard IM NS	0.95–1.1	Comparable	25–40 vs. 10–22	10–15 vs. 8–12	NI; comparable systemic tolerance	DSJI (SC/IM) (SC)	[22]
Hepatitis B (recombinant)	Mixed cohorts	Adolescents/adults	300–800	Anti-HBs GMT	Standard IM NS	0.9–1.3	85–95 vs. 85–98	25–40 vs. 20–35	10–20 vs. 8–18	NI; increased GMT in boosters	DSJI (IM/ID)	[27,28,47]
Rabies (cell-culture)	PEP/PrEP endemic	Children/adults	200–600	Neutralizing Ab titers	Standard NS	1.0–1.4	85–98 vs. 80–95	30–55 vs. 25–50	15–30 vs. 15–32	NI; increased early titers	DSJI (ID/IM)	[27,32,37]
Measles-containing	Mass campaigns	Children	1000–10,000	Seroconversion	Standard SC NS	0.95–1.1	85–95 vs. 83–94	25–40 vs. 20–38	10–20 vs. 10–18	Comparable; increased vaccination capacity	Campaign NFJI (SC)	[22,35,45,47]
mRNA & emerging platforms	Early-phase + modeling	Adults	50–300	Binding/neutralizing Abs	Standard IM NS	0.8–1.2	80–100 vs. 80–100	40–70 vs. 35–65	30–60 vs. 30–60	Feasible; NI pending	Electromech (IM/ID)	[20,23,24,25]

One of the most extensively studied advantages of needle-free jet injections is its ability to support intradermal dose-sparing strategies. The dermis contains a high density of antigen-presenting cells, including dendritic cells and macrophages, which can enhance immune responses when vaccines are deposited accurately within this layer. NFJIs facilitate consistent intradermal delivery by producing controlled lateral dispersion patterns that are less dependent on operator technique than traditional intradermal needle injections [8,13].

### 4.2. Reactogenicity, Pain, and Acceptability

Reactogenicity associated with needle-free jet injection has been evaluated in multiple clinical trials and routine immunization program deployments, with findings largely consistent across vaccine types and delivery routes. Local reactions such as erythema, swelling, induration, and tenderness are commonly reported following NFJI administration, particularly with intradermal delivery. In some studies, the frequency or intensity of local reactions is modestly higher than with needle–syringe injection, reflecting the broader lateral dispersion of fluid within the dermis [12,22,46]. These reactions are typically mild to moderate and resolve spontaneously without clinical intervention. Systemic reactogenicity profiles following NFJI delivery mirror those observed with conventional injections. Fever, malaise, headache, and myalgia occur at comparable rates, indicating that the mechanical mode of delivery does not meaningfully alter systemic immune responses or safety outcomes [27,48]. Importantly, surveillance data from large public vaccination programs have not identified any unique or unexpected adverse-event patterns attributable to jet injection when modern single-use devices are employed [11,26].

Patient acceptability is an important aspect of NFJI use that warrants discussion. Studies examining pain perception show mixed results, affected by injection site, formulation volume, and delivery route. Intradermal jet injections are often described as causing a brief stinging or pressure sensation. In contrast, some recipients find intramuscular jet injections less painful than needle-based injections [12,46]. Fear of needles frequently appears in acceptability studies, with NFJIs often viewed positively by individuals with needle anxiety, especially when devices are accompanied by clear explanations and demonstrations [16,17]. At the public immunization campaign level, acceptability extends beyond individual recipients to include vaccinators and communities. Training studies indicate that vaccinators adapt quickly to NFJI operations and often report improved efficiency in the administration sequence due to reduced sharps handling and simplified preparation steps [11,22]. Community engagement experiences indicate that transparent communication about safety, sensation, and expected local reactions is critical for maintaining trust. When these elements are addressed, NFJIs are generally well accepted and can enhance participation in both routine immunization and mass vaccination campaigns.

### 4.3. Dose-Sparing and Intradermal Immunogenicity

Clinical trials evaluating fractional-dose vaccination have demonstrated that reduced antigen doses delivered intradermally via NFJIs can elicit immune responses comparable to those elicited by full-dose intramuscular injections. In several studies, these fractional intradermal strategies corresponded to approximately 20% of the standard intramuscular dose (that is, a one-fifth dose) while still achieving acceptable serologic responses. This has been demonstrated for the inactivated poliovirus vaccine, where one-fifth intradermal dosing delivered by jet injector supported dose-sparing without loss of immunogenicity in multiple evaluations [28,29,30,32,33,44,45,49]. This effect has also been documented for inactivated influenza, rabies, and other vaccines, with seroconversion rates and antibody titers meeting non-inferiority criteria in multiple studies [19,27,48]. Such findings are particularly relevant during periods of vaccine shortage, where dose-sparing can extend supply without compromising protection. The reproducibility of intradermal vaccine deposition is critical to these outcomes. Studies comparing needle-free jet injectors with manual intradermal needle techniques report lower variability in wheal formation and delivery depth with jet injectors, contributing to more consistent antigen deposition within the dermal layer and more uniform immunogenicity in most recipients [13,46]. Improved reproducibility also reduces reliance on highly specialized injection techniques and limits errors associated with incorrect needle angle or depth during conventional intradermal administration. From a public health immunization perspective, dose-sparing makes both economic and logistical sense. Reduced antigen requirements can lower procurement costs and facilitate rapid scale-up during outbreak responses. However, successful use of NFJIs for fractional dosing requires that injector parameters, formulation characteristics, and immunization protocols remain within validated performance ranges to ensure reliable dose delivery.

## 5. Public Health Vaccination Programs and Operational Experience

### 5.1. Workforce Efficiency and Training

The introduction of needle-free jet injectors into immunization programs has direct implications for workforce efficiency and training requirements. Traditional needle–syringe delivery relies heavily on individual technique, particularly for intradermal injections, where consistent placement demands practice and ongoing skill maintenance. NFJIs reduce this dependency by automating key aspects of delivery, including penetration depth and dispersion geometry, thereby standardizing performance across vaccinators with varying levels of experience [11,22].

Training programs for NFJIs typically focus on device operation, cartridge handling, and basic troubleshooting instead of fine motor injection technique. Field evaluations indicate that vaccinators achieve competency after relatively short training periods, often measured in hours as opposed to days, and retain proficiency with minimal refresher instruction [26,46]. This reduced training burden is particularly valuable in settings with high staff turnover, limited access to formal training infrastructure, or rapid scale-up demands during outbreak responses.

Evidence from large-scale vaccination campaigns indicates that NFJIs can increase the rate of vaccine administration by simplifying preparation and injection procedures. Because the devices eliminate steps such as needle attachment, recapping, and sharps disposal, the vaccination process involves fewer handling steps and reduced task complexity. These features allow vaccination teams to focus on recipient preparation, vaccine administration, and record keeping. In coordinated vaccinations, these efficiencies translate into higher numbers of individuals vaccinated per hour while maintaining safe and accurate dose delivery [12,22]. From a workforce safety perspective, reducing or eliminating sharps handling lowers the risk of accidental needlestick injuries, a persistent occupational hazard in immunization settings. Surveillance reports from programs adopting NFJIs document declines in sharps-related incidents, which contribute to improved worker safety and morale [5,11]. These practical advantages are especially relevant in high-volume immunization campaigns.

### 5.2. Campaign Experience in Middle-Income Countries (LMICs) and Emergency Situations

Needle-free jet injectors have been used in several low- and middle-income countries (LMICs) and emergency immunization settings, providing field evidence of device performance under conditions that challenge conventional needle-based vaccination. Large-scale immunizations, including polio eradication initiatives and measles–rubella vaccination programs, have employed NFJIs to facilitate rapid vaccination of large populations while addressing constraints related to workforce capacity, sharps disposal, and infection control [29,30,32,34,35,36,40,44,45,49]. Clinical and public health vaccination evaluations from regions including South Asia, sub-Saharan Africa, and Latin America have documented the successful deployment of disposable-syringe jet injectors in both routine immunization programs and outbreak response initiatives. These experiences illustrate how NFJIs can support vaccinations in diverse health systems, particularly where workforce constraints, sharps waste management, or supply limitations create barriers to conventional injection methods [29,30,32,34,35,40,44,45,49].

Evidence from low- and middle-income settings highlights several recurring advantages of needle-free jet injectors. Recent immunization initiatives indicate that these devices can support intradermal dose-sparing strategies and rapid vaccination of large populations where conventional needle-based delivery is difficult to sustain. Intradermal fractional-dose inactivated poliovirus vaccine using disposable-syringe jet injectors has demonstrated reliable dermal antigen deposition, acceptable reactogenicity profiles, and strong serologic responses in large public health programs. These findings support the role of NFJIs as practical complements to conventional needle–syringe administration during periods of vaccine supply limitation or rapid expansion of vaccination coverage [29,30,32,33,40,44,45,49]. NFJIs facilitate rapid administration with consistent delivery across diverse populations, including children and adults, even when vaccinators have limited prior experience with intradermal techniques. The elimination of sharps waste simplifies logistics in environments with limited or disrupted waste management infrastructure, reducing the burden on campaign planners and local health systems [22]

Emergency deployments further underscore the value of NFJIs in environments characterized by time pressure, population displacement, or damaged infrastructure. During outbreaks and humanitarian crises, vaccination teams must often operate in temporary clinics or mobile units with limited supplies. NFJIs work well in these situations by minimizing consumables associated with needle–syringe delivery and enabling faster setup and teardown of vaccination stations [11,46]. These features contribute to improved coverage during short intervention windows.

Despite the advantages discussed above, this method of vaccine delivery has challenges. A reliable supply of single-use cartridges and the maintenance of device functionality require coordination, particularly in remote or resource-limited regions. Power requirements for electromechanical systems, as well as import or regulatory hurdles, can further influence feasibility. Nonetheless, evaluations from LMIC campaigns consistently indicate that, when logistics and training are appropriately addressed, NFJIs perform effectively and are well accepted by both vaccinators and communities. These experiences demonstrate that jet injections can be a viable component of immunization strategies in settings where speed, safety, and adaptability are critical.

### 5.3. Community Acceptance and Hesitancy

Community acceptance is a critical determinant of the success of any immunization strategy, and the introduction of needle-free jet injectors can influence perceptions of vaccination in multiple ways. Studies examining community responses to NFJIs consistently identify fear of needles as a significant barrier to vaccine uptake, particularly among children, adolescents, and adults with prior negative injection experiences. By eliminating visible needles, NFJIs can reduce anticipatory anxiety and improve willingness to be vaccinated in these groups [16,17]. Communication and education play a central role in shaping acceptance. Immunization field performance evaluations show that communities respond positively when the function and safety of jet injectors are explained clearly and when recipients are informed about the expected sensation and possible local reactions. Demonstrations and transparent messaging help address concerns related to unfamiliar technology and counter misconceptions that jet injections are more painful or unsafe than conventional methods [22,23]. Perceptions of pain and safety also intersect with broader issues of trust. In some situations, unfamiliar devices initially raised questions about efficacy or intent, underscoring the importance of engaging local leaders, healthcare workers, and community representatives early in campaign planning. Where such engagement occurred, acceptance improved, and hesitancy decreased over time as positive experiences accumulated and were shared within communities [12,46].

Importantly, needle-free jet injectors do not eliminate concerns related to vaccine hesitancy. Their impact is most evident when they are used as part of broader vaccination strategies that address cultural perspectives, access barriers, and public trust in healthcare systems. When supported by clear communication and community engagement, these devices may improve acceptance and participation in vaccination programs.

## 6. Regulatory and Quality Standards

### 6.1. WHO PQS Standards and Device Prequalification

Global adoption of needle-free jet injectors is closely linked to compliance with international regulatory and procurement requirements, particularly the World Health Organization (WHO) Performance, Quality and Safety (PQS) system. WHO PQS prequalification serves as a benchmark for devices intended for use in national immunization programs and for procurement by United Nations agencies. For NFJIs, PQS requirements emphasize consistent delivery performance, infection control safeguards, durability under field conditions, and compatibility with routine immunization administration sequences [46,50].

PQS specifications for injection devices define criteria for pressure generation, dose accuracy, and penetration reliability across the intended routes of administration. Devices must demonstrate that single-use components effectively prevent cross-contamination and that materials in contact with the formulation meet biocompatibility standards. Validation testing includes simulated-use evaluations, environmental stress testing, and performance assessments within temperature and humidity ranges representative of deployment settings [46,50]. Prequalification also considers operational factors relevant to large-scale use. These include ease of user training, comprehensibility of device instructions, mechanical robustness during transport and storage, and compatibility with commonly used vaccine dose formats, vial configurations, and filling volumes. For NFJIs intended for intradermal delivery, PQS assessments examine whether devices reliably achieve the intended deposition depth without excessive variability, supporting consistent immunogenic outcomes [11,22].

WHO PQS listing has practical implications beyond technical validation. Devices that achieve prequalification are eligible for procurement by UNICEF and other international partners, facilitating integration into funded immunization programs. As a result, WHO PQS prequalification has become an important milestone for manufacturers seeking global procurement eligibility and for national immunization programs evaluating NFJIs for routine vaccine delivery.

### 6.2. FDA, EMA, and Health Canada Expectations

Beyond WHO PQS prequalification, needle-free jet injectors must meet the regulatory requirements of national and regional authorities to support clinical use and market access. In the United States, NFJIs are regulated as medical devices and are typically cleared through the FDA 510(k) pathway, which requires demonstrating substantial equivalence to a legally marketed predicate device. Regulatory review focuses on performance testing, biocompatibility, sterility assurance, and verification of single-use safeguards that prevent cross-contamination [46,50].

European regulatory oversight is governed by the Medical Device Regulation (MDR 2017/745) or, for legacy products, the earlier Medical Device Directive. Under these frameworks, NFJIs must obtain CE marking by demonstrating conformity with essential safety and performance requirements, supported by risk management documentation, clinical evaluation, and post-market surveillance plans. Emphasis is placed on device reliability, pressure control, and traceability of single-use components, particularly for devices intended for repeated high-volume use [22].

Health Canada regulates needle-free jet injectors as Class II medical devices under the Medical Devices Regulations (SOR/98-282). Manufacturers must provide evidence of device safety, effectiveness, and compliance with quality management system requirements, typically supported by technical documentation prepared in accordance with internationally recognized ISO standards. Although Health Canada regulatory requirements closely parallel those of the U.S. Food and Drug Administration (FDA) and the European Medicines Agency (EMA), national requirements related to product labeling, bilingual documentation, and post-market surveillance may influence review timelines and market authorization procedures [46,50].

Among regulatory authorities, there is increasing convergence toward standardized expectations for performance validation and risk mitigation. Regulatory agencies frequently reference international standards, such as ISO 21649 [46], for needle-free injectors, thereby promoting consistency in test methods and performance criteria. This convergence reduces duplication of regulatory submissions for manufacturers and provides clearer guidance for procurement agencies and immunization programs evaluating NFJIs for vaccination use.

### 6.3. Verification, Validation, and Post-Market Safety

Verification and validation are central to ensuring that needle-free jet injectors perform reliably within their intended operating range and remain safe throughout their lifecycle. Regulatory expectations require manufacturers to demonstrate that design inputs, such as target pressure profiles, dose accuracy, and penetration depth, are consistently achieved under normal and worst-case conditions. Verification activities typically include bench testing of pressure output, flow characteristics, and mechanical durability, while validation extends to simulated use and, where applicable, clinical performance data [46,50].

Validation protocols place particular emphasis on single-use safeguards and device design elements intended to prevent cross-contamination. Devices must demonstrate effective prevention of backflow and cross-contamination under repeated-use scenarios, even when subjected to user error or environmental stress. Material compatibility and sterility assurance are evaluated to confirm that disposable components maintain integrity throughout storage and use. These requirements reflect lessons learned from earlier generations of jet injectors and underpin contemporary confidence in NFJI safety [4,46,50]. Post-market surveillance complements pre-market testing by capturing real-world performance data after deployment. Manufacturers and regulatory authorities monitor adverse events, device malfunctions, and user feedback to identify trends that may not emerge during controlled testing. In immunization programs, this surveillance is often combined with existing vaccine safety monitoring systems, enabling detection of both device-related and delivery-related issues [11,46]. Continuous feedback from post-market data supports iterative improvements in device design, training materials, and usage guidelines. Regulatory agencies increasingly emphasize proactive risk management and timely reporting to ensure that safety features remain effective as devices are scaled across diverse settings. This ongoing oversight is essential for sustainable use in national immunization programs.

## 7. Economic, Environmental, and Supply Chain Considerations

### 7.1. Cost Drivers in NFJI Deployment

The economic viability of needle-free jet injectors depends on a combination of device-related costs, consumables, and system-level efficiencies. Unlike conventional needle-and-syringe delivery, where unit costs are dominated by inexpensive disposable components, NFJIs require an initial investment in the injector and recurring costs for single-use cartridges or syringes. These factors require evaluation over the full lifecycle of the device, not on a per-injection basis alone [3,30]. Several cost drivers influence overall affordability. Device acquisition costs vary by propulsion type and level of technological sophistication, with mechanically actuated systems generally less expensive than digitally controlled electromechanical device designs. Consumable pricing, including single-use cartridges and protective components, contributes substantially to per-dose costs and is influenced by procurement volume, supply-chain reliability, and regional manufacturing capacity [26,27]. Maintenance, calibration, and replacement cycles further affect long-term budgeting, particularly for devices intended for repeated high-volume use.

These expenditures may be partially offset by downstream cost reductions associated with needle-free administration. NFJIs reduce or eliminate sharps waste, lowering costs related to disposal, transport, and management of needlestick injuries. During large-scale vaccinations, simplified injection procedures and reduced training requirements can translate into lower labor demands and greater vaccination capacity over a given period [11,22]. In addition, dose-sparing strategies enabled by reliable intradermal delivery may decrease vaccine procurement requirements for selected antigens, further enhancing economic efficiency during large immunization campaigns [19,48]. The economic performance of needle-free jet injectors varies depending on the vaccination setting and the conditions under which immunization programs are conducted. In contrast, in low-volume vaccination settings or well-resourced health systems with established sharps management infrastructure, conventional needle–syringe administration may remain economically advantageous. Careful evaluation of these factors is therefore necessary when considering procurement and long-term integration of NFJIs into immunization programs.

### 7.2. Environmental Impact and Sharps Reduction

Environmental considerations increasingly influence decisions around vaccine delivery technologies, particularly in the context of large-scale immunization programs. Conventional needle–syringe delivery generates substantial volumes of sharps waste, including needles, syringes, and contaminated packaging, all of which require secure collection, transport, and disposal. In many settings, inadequate waste management infrastructure increases the risk of environmental contamination and occupational exposure [30,39]. Needle-free jet injectors address some of these challenges by eliminating metal needles and reducing the volume of sharps waste. Routine immunization programs’ assessments indicate that replacing needle–syringe systems with NFJIs can substantially decrease the number of sharps containers required and simplify waste-handling logistics, particularly during mass vaccination campaigns. Reduced sharps waste also lowers the risk of accidental needlestick injuries during waste handling and disposal, thereby improving occupational safety [11,26]. However, NFJIs also involve additional environmental considerations. Single-use cartridges and plastic components generate non-sharps medical waste that must still be managed responsibly. Life-cycle assessments suggest that the environmental impact of these materials depends on factors such as polymer type, manufacturing processes, transportation distances, and end-of-life disposal or recycling options [22]. In some cases, increased plastic waste may offset gains achieved through sharps reduction if disposal systems are not optimized.

Balancing these factors requires a systems-level perspective. Comparative environmental analyses that account for total waste volume, material composition, energy use, and injury risk yield a more comprehensive view of sustainability than sharps reduction alone. As environmental performance becomes a more prominent criterion in procurement decisions, such analyses will be increasingly important in guiding the selection and design of the NFJI system for long-term use.

### 7.3. Procurement and Supply Chain Realities

The scale-up of needle-free jet injectors within immunization programs depends not only on technical performance but also on procurement strategies and supply chain resilience. NFJIs also require device- and consumable-specific logistics that differ from those of conventional needle-and-syringe delivery, thereby influencing how products are sourced, distributed, and maintained. Procurement agencies must consider device availability, compatibility with vaccine types, and the reliability of consumable supply when evaluating NFJI adoption [21,27,30].

Market-shaping efforts by international organizations and donors have played an important role in supporting the deployment of NFJI. Inclusion of prequalified devices in pooled procurement mechanisms and long-term purchasing agreements can stabilize demand and encourage manufacturers to invest in capacity expansion and cost reduction. Such approaches have been used to support the introduction of NFJIs in polio and measles–rubella campaigns, where predictable demand facilitated the timely supply of both devices and cartridges [48,50]. Supply chain considerations extend beyond initial procurement. NFJIs require ongoing access to single-use components, spare parts, and, for some injectors, power sources or charging infrastructure. Disruptions in manufacturing, transportation, or regulatory clearance can affect availability, particularly in regions reliant on imports. Modeling studies highlight the vulnerability of centralized production and the potential benefits of regional manufacturing or diversified sourcing to improve resilience [3,40,45].

## 8. Where the Field Stands Today

### 8.1. Engineering Improvements and Digital Control

Recent developments in needle-free jet injector design reflect broader trends in medical device engineering, particularly the integration of digital control and sensing technologies. Electromechanical NFJIs are increasingly incorporating microprocessors, pressure sensors, and feedback systems that enable real-time alteration of actuation parameters. These capabilities enable finer control over acceleration ramps, peak pressure, and pulse duration, improving injection consistency and expanding the range of formulations and target tissues that can be accommodated [12,24]. Digital control also supports enhanced monitoring and quality assurance. Embedded sensors can record pressure profiles, injection completion, and device status, generating data that confirms proper dose delivery and supports traceability. Such features support regulatory requirements for performance verification and post-market surveillance, particularly where large numbers of injections are administered over short periods [14,26]. From a development perspective, computational modeling and simulation tools are increasingly used to inform NFJI design. Computational fluid dynamics and system-level modeling enable engineers to predict jet behavior, penetration depth, and dispersion patterns under varying conditions, reducing reliance on empirical trial-and-error. When combined with experimental validation, these tools accelerate optimization of nozzle geometry, actuation profiles, and device–formulation compatibility [12,25].

These engineering and digital trends suggest that NFJIs can be adapted to future vaccine delivery needs. Recent investigations have also explored next-generation jet injectors designed to enhance precision and compatibility with emerging biologic formulations. Experimental designs incorporating digitally controlled actuators, microprocessor-regulated pressure modulation, and real-time feedback sensors allow fine adjustment of jet velocity and pulse duration during injection. These capabilities may help optimize delivery for shear-sensitive formulations, such as nucleic acid vaccines, viral vectors, and nanoparticle-based therapeutics. Early studies of programmable and pyro-drive jet injector systems further suggest that tailored pressure profiles can improve intradermal deposition efficiency while maintaining formulation stability [23,24,25].

By enabling precise, data-informed control of jet parameters, next-generation devices may allow delivery conditions to be tailored to specific formulations and dosing strategies, thereby extending the application of needle-free jet injectors beyond their traditional uses. Other minimally invasive vaccine delivery technologies are also being explored, including microneedle-based systems. Microneedle arrays deliver vaccine antigens through microscopic projections that penetrate the stratum corneum while minimizing pain and tissue trauma, in contrast to dissolvable or coated microneedle patches. Needle-free jet injectors deliver liquid formulations at high velocity and may be particularly well suited to vaccination campaigns requiring rapid administration across large populations. These technologies should therefore be considered complementary components within the broader landscape of minimally invasive vaccine delivery strategies.

### 8.2. Device–Vaccine Co-Design and Formulation Integration

As vaccine delivery devices diversify, increasing attention is being paid to the co-design of delivery devices and formulations. For needle-free jet injectors, this approach recognizes that jet formation, pressure profiles, and nozzle geometry interact directly with formulation properties, including viscosity, particle size, and structural stability. Optimizing these elements in isolation can limit performance, whereas coordinated design allows more predictable delivery and improved biologic outcomes [12,30].

Evidence from formulation studies and engineering analyses indicates that matching actuation parameters to formulation tolerance can mitigate shear-induced damage while preserving penetration efficiency. For example, adjusting acceleration ramps or peak pressure allows shear-sensitive lipid nanoparticle or protein formulations to be delivered without compromising integrity, while still achieving consistent intradermal dispersion [21,24]. This consideration is particularly important for nucleic-acid vaccines and complex biologics, where even small variations in mechanical stress can affect molecular integrity and potency. Co-design strategies also extend to vaccine presentation and packaging. Single-use cartridges, prefilled syringes, and integrated delivery units can be engineered to maintain sterility, reduce dead volume, and support accurate fractional dosing. Such an approach simplifies the administration sequence, reduces handling steps, and lowers the risk of user error, benefits that are amplified in high-volume or resource-constrained environments [26,27].

### 8.3. Open Questions for the Next Phase of Jet Injection

Despite substantial progress in needle-free jet injector technology, several unresolved questions remain that will influence future adoption and optimization. One persistent challenge is the limited availability of standardized testing methods that replicate the transient mechanical stresses experienced during jet injection. While pressure-driven and high-shear models are increasingly used, a broader consensus on test protocols would improve comparability between studies and accelerate formulation–device co-development [22]. Another area requiring further investigation is the interaction between jet injection and emerging vaccines. Although available data suggest that many protein-, viral-vectored-, and nucleic-acid vaccines can be delivered safely via NFJIs, systematic evaluations of a broader range of formulations are needed. In particular, understanding long-term stability, subtle structural changes, and batch-to-batch variability under jet injection conditions is crucial [21,24]. Further operational research is needed. For example, many current studies focus on the short-term results of vaccination campaigns, leaving critical questions about long-term device maintenance, injector durability, and cost-effectiveness over extended periods unanswered. Comparative studies evaluating NFJIs using evolving needle-based technologies, including safety-engineered syringes, could provide more detailed evidence for procurement decisions and vaccination program planning [11,22]. Finally, the introduction of digital features raises new questions about data governance, interoperability, and privacy, particularly in environments with limited digital infrastructure. Addressing these issues, together with technical and clinical research, will be important as NFJIs become more connected devices.

## 9. Conclusions

Needle-free jet injectors have regained attention as practical devices for administering vaccines and biologics. Modern devices incorporating single-use fluid pathways and controlled actuation have addressed many of the contamination and reliability concerns associated with earlier multi-use systems and now permit reproducible intradermal and intramuscular administration in clinical settings. Clinical studies show that, for several licensed vaccines, needle-free jet injectors achieve immunogenicity and safety outcomes comparable to conventional needle–syringe delivery while providing practical advantages such as intradermal dose-sparing and reduced sharps waste. Experience from routine immunization programs and emergency vaccination campaigns also highlights operational requirements associated with NFJI use, including personnel training, cartridge supply management, and device maintenance. Further progress will depend on the closer integration of injector engineering with formulation design. Because many modern biologic therapeutics are structurally complex and sensitive to mechanical stress, the interplay among actuation dynamics, formulation stability, and tissue deposition is a critical consideration. Continued refinement of actuation control, improved injector reproducibility, and coordinated device–vaccine development may expand the range of biologics suitable for jet injection while supporting safe and efficient immunization programs.

## Figures and Tables

**Table 1:** Comparison of Needle-Free Jet Injector (NFJI) Device Types.

Device Type	Example Devices	Energy Source	Route/Depth	Typical Volume	Use-Cases	Advantages	Limitations	Citation(s)
Spring-powered NFJI	Biojector; early mechanical jets	Compressed spring	SC or IM	0.1–1.0 mL	Mass vaccination; adult programs	Simple mechanics; good reliability	Recoil, noise; historical cross-contamination in multi-use nozzles	[26,27,28,29]
Gas-powered NFJI	Ped-O-Jet; legacy WHO devices	CO_2_/Nitrogen	SC or IM	0.1–1.0 mL	Smallpox/measles historical programs	Adjustable pressure; vaccination of large population;	Bulky gas cylinders: maintenance burden	[13,22,30]
Disposable-Syringe Jet Injectors (DSJI)	PharmaJet Stratis/Tropis; single-use syringe	Spring or gas	ID/IM/SC	0.1–0.5 mL	Campaign vaccination; routine immunization	Reduced cross-contamination risk; easier field hygiene	Higher per-dose cost; training required	[2,11,17,19,31]
Electromechanical NFJI	Digital motor-controlled systems	Electromechanical actuator	ID/IM (programmable)	0.05–0.5 mL	Precision ID delivery; biologics	Programmable pressure profiles; better dose control	Higher cost; complex regulatory path	[10,13,15]
Microjet/Microarray Jet Systems	Micro-jet arrays; hybrid NFJI–microneedle devices	Mechanical or EM	Dermal/mucosal	≤0.1 mL	ID vaccines; mucosal delivery	Very low pain; strong dose-sparing potential	Limited clinical maturity; complex manufacturing	[19,32]
Mucosal Jet Injectors	Nasal/oral jet systems	Pneumatic or spring	Nasal/oral mucosa	0.1–0.5 mL	Influenza; pediatric mucosal vaccines	Non-invasive; high acceptability	Formulation constraints; variable deposition patterns	[8,14,23]

## Data Availability

No new datasets were generated during this study. All data supporting the findings of this review are derived from previously published studies and are appropriately cited within the article.

## Abbreviations

The following abbreviations are used in this manuscript:

**Abbreviation**	**Definition**
NFJI	NFJI—Needle-Free Jet Injector
WHO	World Health Organization
PQS	Performance, Quality and Safety (WHO device prequalification framework)
FDA	U.S. Food and Drug Administration
EMA	European Medicines Agency
HC	Health Canada
LMICs	Low- and Middle-Income Countries
IM	Intramuscular
ID	Intradermal
SC	Subcutaneous
mRNA	Messenger Ribonucleic Acid
CFD	Computational Fluid Dynamics
CCI	Container Closure Integrity
CQA	Critical Quality Attribute
CPP	Critical Process Parameter
GMP	Good Manufacturing Practice
OOS	Out-of-Specification
PPE	Personal Protective Equipment
AE	Adverse Event
AEFI	Adverse Event Following Immunization
R&D	Research and Development
LC	Liquid Chromatography
HPLC	High-Performance Liquid Chromatography
USP	United States Pharmacopeia
ICH	International Council for Harmonisation
GDP	Good Distribution Practice
COC	Chain of Custody
CE	Conformité Européenne (European conformity certification)
EUA	Emergency Use Authorization
ICT	Information and Communication Technology
GMT	Geometric Mean Titer (immunologic antibody concentration metric used in vaccine studies)
NI	Non-Inferiority (clinical trial comparison showing a new intervention is not worse than the standard by a predefined margin)
